# A narrative review of the last decade’s literature on the diagnostic accuracy of septic arthritis of the native joint

**DOI:** 10.1186/s40634-020-00315-w

**Published:** 2021-01-09

**Authors:** Elizabeth H. G. Turner, Mc Daniel H. Lang, Andrea M. Spiker

**Affiliations:** Department of Orthopedics and Rehabilitation, University of Wisconsin - Madison, UW Health at The American Center, 4602 Eastpark Blvd., Madison, WI 53718 USA

**Keywords:** Septic arthritis, Septic joint, Joint aspiration

## Abstract

While septic arthritis can be a straightforward diagnosis, there are many cases when the diagnosis is difficult to make. The aim of this study was to review the last decade’s literature on the diagnosis of septic arthritis of the native joint in adults and summarize that data in an easy to follow algorithm to clarify how the last decade’s data may be applied to the diagnosis of septic arthritis. A search of PubMed and CINAHL databases was performed to identify studies that compared results diagnostic tests for septic arthritis. We cross referenced this search with searches of additional databases (including Cochrane Library and Scopus) to confirm similar search results. The Quality Assessment of Diagnostic Accuracy Studies (QUADAS) tool was used by two independent reviewers to determine study quality and risk of bias. After applying inclusion and exclusion criteria to the initial search, 15 papers total were included for analysis. All 15 papers were of high quality methodology as determined by the QUADAS tool. There were 26 different diagnostics tests used across the 15 papers included for review. Three of those diagnostic tests had specificity and sensitivity greater than 80%. Eight tests had a positive likelihood ratio of ≥10. Three tests had a negative likelihood ratio < 0.1, indicating that they may help to rule out septic arthritis. A flowchart was created to summarize the findings of our review, so that physicians may reference this visual in making the appropriate diagnosis when the commonly held standards of cell count, gram stain, and culture aren’t enough to make the diagnosis.

## Introduction

The diagnosis of septic arthritis primarily relies on patient clinical presentation and synovial fluid analysis of the affected joint. The differential diagnosis of septic arthritis is broad and may include gout, pseudogout, trauma, hemarthrosis, rheumatic fever, rheumatoid arthritis, spondyloarthropathies, osteomyelitis, viral arthritides, septic bursitis, and lyme disease [7]. While synovial fluid analysis is obtained as standard of care, it is well-document that synovial fluid findings can be highly variable and there can be significant overlap in patients with the underlying diagnosis of gout, pseudogout, or rheumatoid arthritis [9, 30]. A synovial white blood cell (WBC) count of 50,000 has typically been used as an appropriate cut-off for the diagnosis of septic arthritis, though gout and pseudogout are known to result in similar WBC counts [23]. Conversely, some patients with septic arthritis may have WBC counts < 50,000, and the immunosuppressed patient may mount little to no leukocytic response at all. Septic arthritis can also coexist with crystalline arthropathy, thus further confounding the diagnosis of septic arthritis [7, 22]. Septic arthritis, gout, and pseudogout are known to have elevated serum inflammatory markers such as erythrocyte sedimentation rate (ESR) and C-reactive protein (CRP), rendering them of little use in differentiating the diagnosis of septic arthritis from systemic disease. Similarly, serum glucose and protein have failed to show diagnostic utility [9]. Recent analysis has shown some promise for the use of other serum markers such as procalcitonin levels, the delta neutrophil index, calprotectin levels, and the lactate/glucose ratio [3, 5, 21]. Culture and gram stain can help to confirm septic arthritis, though these can be falsely positive due to contamination from skin flora. Colony counts and sensitivities can help to confirm actual infection vs. contamination. Alternatively, a negative culture can occur in septic arthritis due to initiation of antibiotics prior to synovial fluid sampling, inadequate fluid sampling volume, or inadequate plating and growth requirements. All of this points to the difficulty that can arise in properly diagnosing septic arthritis and the need for a synopsis of recent literature on the topic of the diagnostic accuracy of septic arthritis.

The aim of this study was to review the last decade’s literature on the diagnosis of septic arthritis of the native joint in adults and summarize that data in an algorithm in order to clarify how the last decade’s literature may be applied to the diagnosis of septic arthritis.

## Methods

### Study design

This narrative review was performed according to the criteria of the Preferred Reporting Items for Systematic Reviews and Meta-Analysis (PRISMA) recommendations [14].

### Search strategy

A systematic search of relevant literature was conducted using PubMed and CINAHL, with cross referencing total manuscript counts in the Cochrane Library and Scopus, as well as hand-searching reference lists of included articles. Search terms used were “septic arthritis”, “septic joint”, “diagnosis”, “approach”, and “synovial fluid”. We subsequently re-ran the search with additional terms such as “infection” “infectious” and confirmed that our results were similar. Results were then narrowed to include only the adult population, native joint infections, and literature from the last 10 years.

### Study selection

**Fig. 1:**
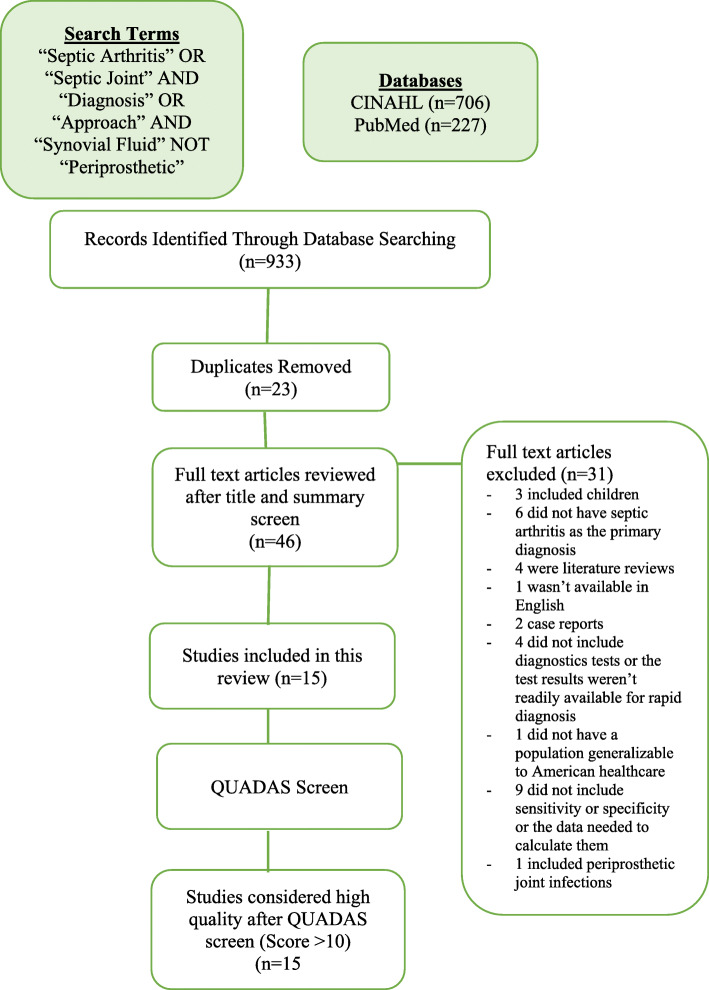
Study selection

A total of 933 articles were reviewed by the first author to identify studies related to the diagnostic accuracy of clinical tests in septic arthritis of the native joint in the adult population. Titles and abstracts were initially reviewed for a primary screen. Full text articles were retrieved during further screening for inclusion (Fig. 1).

### Eligibility criteria

Diagnostic studies were eligible if they included 1) a description of the clinical approach to diagnosing septic arthritis; 2) an assessment of the accuracy of their diagnostic test (e.g, sensitivity, specificity, positive and negative likelihood ratios, or odds ratios) 3) an acceptable reference standard for comparison; 4) were written in the English language; and 5) conducted their study on the adult population with native joints. Studies that included pediatric patient populations were excluded as more formalized diagnostic criteria already exist for some cases of pediatric septic arthritis, such as the Kocher criteria. Patients with prosthetic joints were not included in this review. Studies were excluded if they did not provide an adequate reference standard or a report of diagnostic accuracy.

### Data extraction

Data extraction was performed by the first author. Data extracted included: study population, types of arthritides included, studied diagnostic test, diagnostic reference standard, diagnoses made by the authors, sensitivity, specificity, positive likelihood ratios, and negative likelihood ratios, when available. In studies where odds ratios were provided, a quadratic formula described by Simel et.al. was used to back-calculate sensitivity, specificity, and likelihood ratios if positive and negative test result data was available [27]. Due to heterogeneity amongst outcome measures and diagnostic methods, outcome measures and data could not be combined into a summary set of measures for meta-analysis to create overall diagnostic outcome effects.

Sensitivity, defined as the ability of a test to correctly classify a person with the disease of interest as having that disease, was calculated as (true positives/(true positives + false negatives)). Specificity, defined as the ability to correctly classify a person without the disease of interest as not have that disease, was calculated as (true negatives/(true negatives + false positives)). A likelihood ratio, defined as the likelihood of a given test result in a person with the disease of interest compared with the likelihood of the same result in a person without the disease of interest, was also calculated. A positive likelihood ratio was calculated as (sensitivity/(1-specificity)). A negative likelihood ratio was calculated as ((1-sensitivity)/specificity). Sensitivity and specificity were considered to be sufficient if they were both >/= 90%. A likelihood ratio greater than 10 indicated that a positive test was good at ruling in a diagnosis, while a likelihood ratio less than 0.1 indicated that a negative result was good at ruling out a diagnosis [8, 13].

### Quality assessment

Articles included in the study were assessed for potential bias and quality using the Quality Assessment of Diagnostic Accuracy Studies (QUADAS) tool. This is a 14-question tool with each question scoring 1 point for a “yes” answer and 0 points for a “no” or “unclear” answer. Papers that received a score of 10 or above were considered high quality with less risk of design bias, while those which received a score below 10 were considered low quality with higher risk of design bias. Two reviewers (EHGT and MHL) independently assessed each paper using the QUADAS tool. Disagreements between reviewers were discussed and resolved.

### Level of evidence

The level of evidence was assessed using the Knee Surgery, Sports Traumatology, Arthroscopy level of evidence table provided to authors at: https://www.kssta.org/authors-homepage/level-of-evidence/.

### Source of funding

None.

## Results

### Systematic search results and study selection

A total of 933 articles were retrieved after the initial searches (Search 1: 706 articles, Search 2: 227 articles). The initial 933 were reviewed by title and summary and narrowed to 46. These 46 papers were reviewed via full text and narrowed to 15 after removing studies that included children, periprosthetic joint infections, tests that would not be readily available to aid in the rapid diagnosis of septic arthritis in the hospital setting, did not have the diagnosis of septic arthritis as the primary diagnosis, or did not have sensitivity, specificity, or positive and negative result data needed to compare the diagnostic accuracy of their results. Figure 1 provides a flow diagram describing study selection.

### Characteristics of included studies

Table 1 provides details on study characteristics. Diagnostic tests investigated, participants, diagnostic reference tests used, and diagnoses made by investigators were included. There were 26 different diagnostic tests used across 15 papers included in this review. These tests assessed a total of 1997 patients.

**Table 1:** Characteristics of Included Studies

Author	Test Evaluated	Subjects (N, Gender, Mean age)	Reference Standard
Berthoud (2020) [3]	Lactate, Glucose, Lactate/Glucose Ratio	233 151 males mean age 61.2	Newman’s Criteria **and** 1) pathogen isolated from synovial fluid 2) pathogen isolated from blood culture with typical clinical presentation for arthritis **or** 3) arthrocentesis revealed purulent synovial fluid in combination with presence of typical clinical presentation for septic arthritis, absence of crystals, and absence of other suitable diagnosis
Sigmund (2019) [26]	mPCR, synovial culture	72 39 males mean age 64	1) Newman’s Criteria **or** 2) Pathologic features of septic arthrtitis and leukocyte count > 50,000 or PMNs > 90% in synovial fluid
Chouk (2019) [5]	WBC, CRP, Procalcitonin	98 51 males mean age 65.2	Newman’s Criteria
Shu (2019) [25]	Synovial lactate	39 28 males mean age 51	1) Positive synovial fluid culture **or** 2) Septic arthritis diagnosed by institution orthopedists with surgical intervention and IV antibiotics given during hospital stay
Baillet (2019) [1]	Calprotectin	74 38 males mean age 70.4	Bacteria in synovial culture or blood culture without crystals present
Morgenstern (2018) [15]	SF leuk w/ diff, PCR, and microcalorimetry	57 31 males mean age 62	Positive synovial fluid culture **or** 1) local clinical signs and symptoms, 2) increased SF leukocyte count and 3) exclusion of noninfectious causes
Pavic (2018) [20]	Highest recorded temp w/I 24 h, hemoglobin, WBC, platelet, CRP, ESR, Urate, Syn total leuk, Syn total eryth, syn tot polymorphos, syn tot mononucs, gender, symptom duratioin, features of sepsis, presence of sweats, presence of chills/rigors, joint swelliing, joint erythema, joint warmth, joint tenderness to palpatoin, joint ROM restriction	165 119 males mean age 59.35	Positive synovial fluid culture
Ferreyra (2017) [6]	ALC, ANC, neutrophil differential, crystals	208 125 males mean age 59.6	Pyogenic organism in joint fluid or blood samples
Borzio (2016) [4]	ESR, serum WBC, syn WBC, neutrophils, lymphocytes, temp	458 (gender, age not given)	Positive synovial fluid culture
Paosong (2015) [18]	Procalcitonin	75 no gender, age given	Newman’s Criteria
Lenski (2014)1 [9]	IL6, Synovial Lactate	119 54 males mean age 69.9	Positive synovial fluid culture
Omar (2014) [17]	Leukocyte Esterase and Glucose Strips	146 64 males mean age 59	1) A pathogen was isolated from the synovial fluid 2) A pathogen was isolated from a source other than synovial fluid and the clinical presentation was typical of septic arthritis **or** 3) The synovial fluid was turbid, and crystals were absent.
Baran (2014) [2]	WBC, %PMNs	96 59 males mean age 47	Positive synovial fluid culture
Lenski (2014)2 [9]	Serum markers (WBC, CRP, UA), synovial markers (lactate, glucose, UA, LDH, WBC, tot prot, IL 6)	82 47 males mean age 72.4	Positive synovial fluid culture
Talebi-Taher (2013) [28]	serum/synovial procal, serum IL-6, TNF-a, CRP, ESR, synovial WBC and PMN %	75, 41 males mean age 52.2	1) Purulent material in the joint space with isolation of a bacterial pathogen from the joint fluid **or** 2) Positive gram stain

The most common reference standard used for a diagnosis of septic arthritis was a positive synovial fluid culture. Many studies also used Newman’s criteria as their reference standard [16]. Some studies used either Newman’s criteria or a positive synovial fluid culture or a combination of clinical presentation, synovial fluid leukocyte counts, and the exclusion of other noninfectious causes. One study used either positive synovial fluid cultures or a diagnosis of septic arthritis by an orthopedic surgeon with surgical intervention and intravenous antibiotics given during the hospital stay.

Sizes of study populations between papers ranged from 46 patients to 458.

The different diagnostic tests included synovial lactate, synovial glucose, synovial lactate:glucose, PCR, synovial culture, synovial white blood cell count (WBC) with and without differential, c-reactive protein, procalcitonin, synovial calprotectin, microcalorimetry, urate, synovial total protein, synovial erythrocytes, synovial total polymorphic cells, synovial total mononuclear cells, hemoglobin, platelet counts, absolute neutrophil counts, neutrophil differential, crystals, interleukin-6, leukocyte esterase and glucose test strips, lactate dehydrogenase, and tumor necrosis factor alpha.

### Diagnostic accuracy

The diagnostic accuracy of the clinical tests studied, calculated by sensitivity, specificity, and positive and negative likelihood ratios are provided in Table 2. Sensitivity ranged from 23% - 100% and specificity ranged from 3.5% - 100%. Positive likelihood ratios ranged from 0 to 111.88 and negative likelihood ratios ranged from 0 to 2.23. Three diagnostic tests had both sensitivity and specificity >90%; these were synovial leukocytes > 50,000 or PMNs > 90% (94%, 100%), leukocyte esterase ++ or +++ and glucose – (89.5% and 99.2%), and PMNs > 75% (100%, 94%). Eight tests had a positive likelihood ratio of ≥ 10. These were lactate:glucose ratio > 5 (LR+ 27), synovial lactate >/= 10 (LR+ 41.6), synovial glucose < 1.0 (LR+ 33.3), calprotectin > 150 mg/L (LR+ 12.2), neutrophils > 95% in the absence of crystals (LR+ 11.36), leukocytes > 50,000 in the absence of crystals (LR+ 10.94), leukocyte esterase ++ or +++ and glucose – (LR+ 111.88), and PMNs > 75% (LR+ 16.67).

**Table 2:** Diagnostic Accuracy of Included Tests

	Test	Sensitivity	Specificity	NPV	PPV	LR+	LR-
Berthoud	Lactate/Glucose Ratio > 5	52.0%	98.10%			**27**	0.49
	Synovial Lactate >/=10	40.0%	99.00%			**41.6**	0.61
	Synovial Glucose < 1.0	32.0%	99.00%			**33.3**	0.69
Sigmund	SF mPCR	38.0%	100.00%	100	53.6	0	0.62
	Sf Culture	29.0%	100.00%	100	50	0	0.71
	Combined culture + mPCR	43.0%	100.00%	100	55.6	0	0.57
	Tissue Culture	40.0%	100.00%			0	0.6
Chouk	PCT > 0.5 ng/ml	65.0%	91.00%	65	91	7.20	0.40
	PCT > 0.2 ng/ml	80.0%	74.40%	44.4	93.5	3.10	0.30
Shu	Synovial Lactate >/=10	27.0%	97.00%			7.90	0.80
	Synovial Lactate >/= 5	55.0%	76.00%			2.30	0.60
	WBC >/= 50,000	27.0%	97.00%			7.90	0.80
	WBC >/=100,000	18.0%	100.00%			**10.00**	0.80
Baillet	Calprotectin > 150 mg/L	73.0%	94.00%	84	88	**11.64**	0.29
	Calprotectin < 52 mg/L	96.0%	44.00%	47	95	1.71	**0.09**
Morgenstern	Serum CRP > 10	78.0%	40.00%	48	71	1.30	0.55
	Serum WBC > 10	62.0%	74.00%	65	71	2.38	0.51
	SF Leuks > 50,000 or PMNs > 90%	**94.0%**	**100.00%**	100	97	0.00	0.06
	Culture	46.0%	100.00%	100	74	0.00	0.54
	PCR	23.0%	91.00%	63	65	2.56	0.77
	Microcalorimetry	46.0%	94.00%	83	73	7.67	0.57
Pavic	Features of Sepsis (RR > 25 bpm, HR > 120 bpm, SBP < 100, temp > 38.5 w/I 24 h of review)	75.0%	72.10%	24.7	93.5	1.93	0.41
Ferreyra	Neutrophil count > 50,000	50.0%	94.40%			8.93	0.53
	Neutrophil count < 15,000	92.3%	77.00%			4.013	**0.10**
	Leukocyte count > 50,000	53.6%	91.70%			5.76	0.51
	Leukocyte count > 70,000	39.3%	95.60%			8.93	0.63
	Leukocyte count< 20,000	92.3%	70.60%			3.14	0.11
	%Neutrophils> 90	71.4%	79.70%			3.52	0.36
	%Neutrophils> 95	50.0%	89.00%			4.55	0.56
	%Neutrophils< 80	96.2%	56.00%			2.19	**0.07**
	%Neutrophils> 90 + no crystals	67.9%	92.80%			8.28	0.35
	%Neutrophils> 95 + no crystals	50.0%	95.60%			**11.36**	0.52
	> 50,000 leukocytes + no crystals	53.6%	95.10%			**10.94**	0.49
	> 50,000 neutrophils + no crystals	35.7%	96.70%			**10.82**	0.66
Borzio	Synovial fluid WBC > 64,000	40.0%	90.00%			4	0.25
	Kocher criteria + synovial WBC > 64,000	0.0%	98.60%	0	90.8	0	1.01419878
Paosong	Procalcitonin >/= 0.66	59.0%	86.00%	69.9	79.6	4.21	0.48
Lenski1	Synovial tot prot 4.3	55.6%	75.00%			2.22	0.59
	Syn Gluc 40	56.6%	83.00%			3.33	0.52
	Synovial Lactate 6.2	74.5%	87.20%			5.81	0.29
	Syn WBC 14.4	71.2%	84.90%			4.71	0.34
	Synovial IL6 7000	92.5%	64.10%			2.58	0.12
Omar	LE ++ or +++	94.7%	73.20%	34.6	98.9	3.54	**0.08**
	LE ++ or +++ and GLC -	**89.5%**	**99.20%**	94.4	98.4	**111.88**	0.11
Baren	WBC > 50,000	72.7%	92.30%			9.09	0.30
	% PMNs> 90	81.8%	67.30%			2.50	0.27
	%PMNs> 85	88.6%	57.70%			2.05	0.23
	%PMNs> 80	93.2%	53.80%			2.32	0.11
Lenski2	Synovial lactate >/= 4.3	89.5%	77.30%			3.94	0.14
	Synovial Glucose< 51.5	65.9%	92.00%			8.24	0.37
	(Gout) (synovial uric acid of 7.0)	78.1%	82.80%			4.53	0.27
	(Gout) (Serum uric acid of 7.2)	70.00%	85.20%			4.73	0.35
	Synovial LDH>/=1900	68.9%	88.90%			6.2	0.35
	Synovial WBC>/=38.0	58.2%	86.20%			4.22	0.49
	Synovial total prot of>/=4.4	48.90%	75.00%			1.96	0.68
	Synovial IL-6 of>/=7000	93.90%	13.60%			1.09	0.44
	Serum CRP of>/=0.5	92.30%	3.50%			0.96	2.23
	Peripheral WBC>/=10.0	55.80%	41.40%			0.95	1.07
Talebi-Taher	WBC > 50,000	100%	66%	59.52	100	2.94	0.00
	PMN > 75%	**100.00%**	**94.00%**	89.29	100	**16.67**	0.00
	CRP > 18 mg/L	92.00%	30%	76.47	79.31	1.31	0.27
	ESR (> 17 for men, > 25 for women)	100.00%	26.00%	40.32	100	1.35	0.00
	TNF-alpha > 10	96.00%	62%	55.81	96.88	2.58	**0.06**
	IL6 > 20	12.00%	92%	42.86	67.65	1.50	0.96
	Serum PCT > 0.5 ng/ml	68.00%	80.00%	62.96	83.33	3.40	0.40
	Synovial fluid PCT > 0.5%ng/ml	24.00%	96%	75	71.64	6.00	0.79

### Quality scores

The quality scores of the manuscripts as assessed by the QUADAS tool ranged from 10 to 13, indicating that all of the papers were high quality with low risk of design bias (Table 3). There was 93% initial agreement using QUADAS scores and 100% after further discussion.

**Table 3:** QUADAS Tool Scoring and Level of Evidence (LOE)

Author	Q1	Q2	Q3	Q4	Q5	Q6	Q7	Q8	Q9	Q10	Q11	Q12	Q13	Q14	TOTAL	LOE
Berthoud (2020) [3]	1	1	1	1	1	1	1	1	1	0	0	1	1	1	12	Diagnostic II
Sigmund (2019) [26]	1	1	1	1	1	1	1	1	1	1	0	1	1	1	13	Diagnostic II
Chouk (2019) [5]	1	1	1	1	1	1	1	1	1	0	0	1	0	0	10	Diagnostic II
Shu (2019) [25]	1	1	1	1	1	1	1	1	1	0	0	1	0	0	10	Diagnostic II
Baillet (2019) [1]	1	1	1	1	1	1	1	1	1	0	0	1	0	0	10	Diagnostic II
Morgenstern (2018) [15]	1	1	1	1	1	1	1	1	1	0	0	1	1	1	12	Diagnostic II
Pavic (2018) [20]	1	1	1	1	1	1	1	1	1	0	0	1	0	1	11	Diagnostic III
Ferreyra (2017) [6]	1	1	1	1	1	1	1	1	1	0	0	1	0	1	11	Diagnostic III
Borzio (2016) [4]	1	1	1	1	1	1	1	1	1	0	0	1	0	1	11	Diagnostic III
Paosong (2015) [18]	1	1	1	1	1	1	1	1	1	0	0	1	0	0	10	Diagnostic II
Lenski (2014)1 [9]	1	1	1	1	1	1	1	1	1	1	0	1	0	0	11	Diagnostic III
Omar (2014) [17]	1	1	1	1	1	1	1	1	1	1	0	1	0	1	12	Diagnostic II
Baran (2014) [2]	1	1	1	1	1	1	1	1	1	0	0	1	0	1	11	Diagnostic III
Lenski (2014)2 [9]	1	1	1	1	1	1	1	1	1	0	0	1	0	0	10	Diagnostic III
Talebi-Taher (2013) [28]	1	1	1	1	1	1	1	1	1	0	0	1	0	0	10	Diagnostic II

### Level of evidence

The level of evidence as assessed using the Knee Surgery, Sports, Traumatology, Arthroscopy table provided to authors indicated that all the papers were of either level II (60%) or level III (40%) evidence, in the Diagnostic category (Table 3).

## Discussion

While septic arthritis is sometimes easily diagnosed, there are many situations in which the diagnosis can be confounded by underlying or concomitant disease processes or pathologies. Our goal was to review the last decade’s literature on the diagnosis of septic arthritis of the native joint in adults and provide an algorithm summarizing the findings of the last decade into an easy to follow workflow. The typical patient presentation consists of an acute onset of joint pain with erythema, warmth, limited joint range of motion, and possible effusion within the joint [7]. The first step in diagnosing any suspected septic arthritis is to perform a joint aspiration for gram stain, culture, and cell count of the aspirated synovial fluid, prior to the administration of antibiotics. Successful aspiration of various joints requires knowledge of the anatomical structures that could preclude access to the joint space. When aspirating the ankle, the needle should be inserted 2.5 cm proximally and 1.3 cm anteriorly to the tip of the lateral malleolus, just lateral to the peroneus tertius tendon. The knee can be aspirated on the lateral side, at the superior aspect of the patella. The needle must be advanced through the lateral retinaculum to enter the joint. The hip can be aspirated from either a lateral, medial, or anterior approach. It may be necessary to use advanced imaging to aid in needle placement within the hip capsule. The shoulder is most easily aspirated anteriorly, where the bony landmarks are readily palpable. The needle should be inserted at half the distance between the coracoid process and the anterolateral edge of the acromion, with the needle directed posteriorly so as to avoid the neurovascular bundle of the brachial plexus. The elbow is typically aspirated via a posterior approach, with the needle inserted just lateral to the olecranon [11] (Table 4). Previous literature has shown that the commonly held threshold of synovial WBC > 50,000 is not sensitive enough to effectively rule out septic arthritis [10, 12]. It is also well described that other arthritides such as gout, pseudogout, or rheumatoid arthritis can cause a high synovial WBC count as well [23]. Notably, there are also many cases of concurrent bacterial infection along with crystalline arthropathy, so the presence of crystals isn’t sufficient to rule out bacterial invasion [19, 24, 29]. We performed a broad systemic review of strategies to diagnose septic arthritis in the adult population. Many studies report differing approaches to the diagnosis of septic arthritis using varying lab criteria. Pooled analysis of outcome measures was not feasible due to broad heterogeneity amongst diagnostic approaches.

**Table 4:** Characteristics of Common Joint Aspirations

Joint	Approach	Typical Fluid Amount^**a**^ (mL) [21]	Additional Notes
**Shoulder**	Anterior, at half the distance between coracoid and anterolateral edge of acromion	40–60 ml	Aim needle posteriorly and err laterally to avoid neurovascular bundle of the brachial plexus
**Elbow**	Posterior, just anterolateral to the acromion	20–30 ml	Aim the needle medially and anteriorly
**Hip**	Lateral/medial/or anterior	5–12 ml [15]	Utilize ultrasound to guide the needle into the area to be drained
**Knee**	Lateral, at the superior pole of patella	100–200 ml	The needle should “pop” through the lateral retinaculum before you enter the joint space
**Ankle**	Just lateral to peroneus tertius tendon	20–30 ml	Posterior approach is not recommended as it can damage the articular surface

^a^Determined via saline loading

This review confirms that for any suspected septic arthritis the first step should be aspiration for cell count, gram stain and culture. However, as gram stain and culture take time and the progression of joint destruction due to bacterial invasion can be quite rapid, this review highlights a number of other diagnostic approaches a clinician can take to more quickly arrive to the proper diagnosis. Of the manuscripts reviewed, three tests had both excellent sensitivity and specificity (> 90%) and could potentially serve as rapid diagnostic tests while waiting for a culture. They were synovial leukocytes > 50,000 and/or PMNs > 90%, leukocyte esterase ++ or +++ and glucose –, and PMNs > 75% [15, 17, 28]. Notably, other studies have argued that synovial leukocytes > 50,000 is not sufficiently sensitive to rule out a diagnosis of septic arthritis. Other tests had excellent likelihood ratios of > 10, indicating that they could aid in the diagnosis as a confirmatory “rule in” test, after synovial cell counts were already collected. The tests with positive likelihood ratios > 10 included lactate:glucose ratio > 5, synovial lactate ≥ 10, synovial glucose < 1.0, calprotectin > 150 mg/L, neutrophils > 95% in the absence of crystals, leukocytes > 50,000 in the absence of crystals, leukocyte esterase ++ or +++ and glucose –, and PMNs > 75% [1, 3, 6, 17]. Of these tests presented, serum lactate and glucose are easily drawn labs, readily available in the vast majority of hospital settings. A dipstick for synovial leukocyte esterase and glucose is also an easily available test, with an excellent positive likelihood ratio. While Ferreyra et al. detailed the ways in which the absence of crystals and a high leukocyte or neutrophil count can help to confirm the diagnosis of septic arthritis, the presence of crystals cannot effectively rule out septic arthritis, as described earlier [6, 29].

To that end, several tests had good negative likelihood ratios (LR- ≤ 0.1) and may help to more quickly rule out septic arthritis. Baillet et al. demonstrated that a synovial calprotectin of < 52 mg/L was a highly sensitive test (96%) with a LR – of 0.09 and Ferreyra et al. found that a synovial neutrophil count of < 15,000 had a sensitivity of 92% with a LR- of 0.10 [1, 6]. Lastly, Talebi-Taher et.al. described serum TNF-alpha as an excellent discriminator between septic and inflammatory arthritis with a sensitivity of 96%, a negative predictive value of 96.88%, and a negative likelihood ratio of 0.06. These additional studies may help to rule out septic arthritis in more difficult to diagnose patients with synovial WBC counts less then 50,000.

**Fig. 2:**
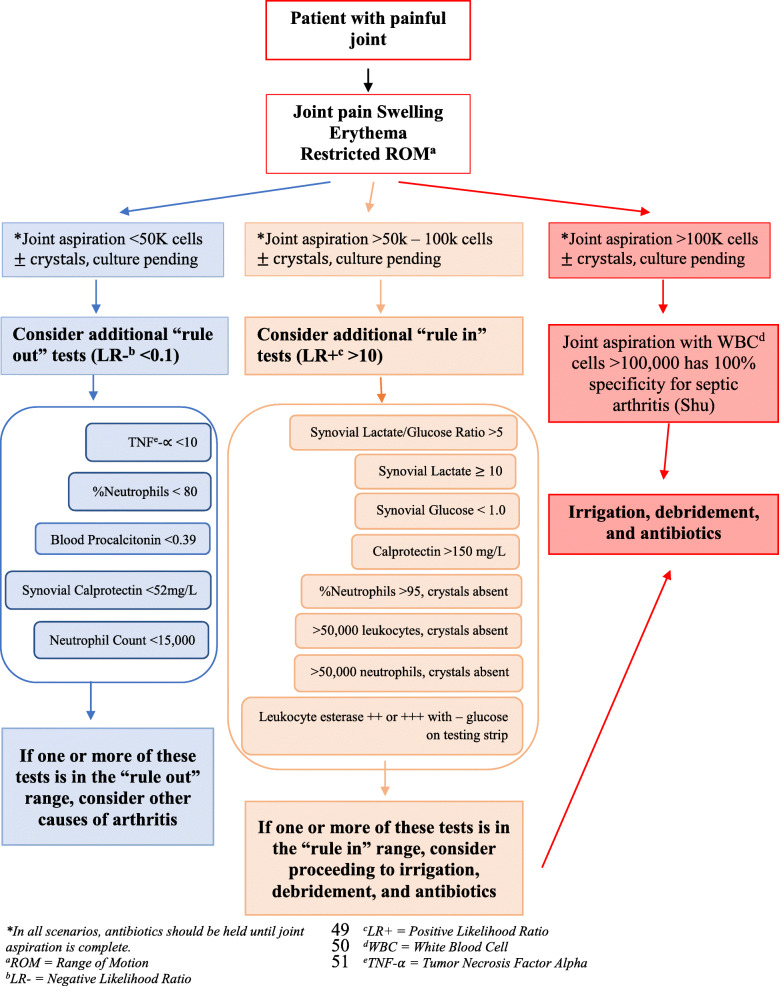
Algorithm summarizing findings of this review

From the information synthesized above we have created an evidence-based algorithm summarizing our findings that practitioners may use to more rapidly narrow the diagnosis of septic arthritis while waiting for gram stain and culture to return (Fig. 2). The literature review confirms that for any suspected septic arthritis joint aspiration with synovial fluid analysis consisting of cell count, gram stain, and culture be performed. While awaiting culture results, additional tests based on the algorithm in Fig. 2 may be utilized to aid in “ruling in” or “ruling out” the diagnosis of septic arthritis, depending on the synovial cell counts.

### Limitations

The main limitation in this review is the absence of a universally accepted gold standard for the diagnosis of septic arthritis. While many papers cited a positive synovial fluid culture as their reference standard, others referred to clinical criteria or a clinical course consisting of surgical intervention and antibiotics. The heterogeneity of diagnostic tests as well as the varying population sizes included in the studies makes it difficult to create uniformly generalizable conclusions about the diagnosis of septic arthritis. Additionaly, laboratories at various institutions may have different ‘normal ranges’ and therefore the numbers presented may not be universal measurements, but may need to be converted into differing institutional ranges. This review is further limited by the small number of articles published about the potential diagnostic accuracy of tests in septic arthritis. The narrow field may have introduced bias into our review. Language limitations of the reviewers narrowed the acceptable studies to those published in English. Lastly, while an attempt was made to assess the potential for bias and quality using the QUADAS tool, this tool does have its own limitations. A paper may score poorly on the QUADAS tool if certain methodological steps are not included in the manuscript, even if they were part of the analysis. This field would further benefit from high quality methodological research to further narrow the diagnostic potential of some of the referenced biomarkers in the approach to septic arthritis.

## Conclusions

This narrative review aggregates and synthesizes the last decade of published literature on the approach to diagnosing septic arthritis in the adult native joint, and we have provided a visual algorithm summarizing our review. The initial step in diagnosis is a joint aspiration with culture and gram stain. Notably, culture and gram stain are still the gold standard when it comes to accurately diagnosing septic arthritis of the native joint, however, while culture and gram stain are pending, there are additional tests that can help to either rule in or rule out septic arthritis if the preliminary cytology is not overly convincing. Test findings such as TNF-alpha < 10, percent neutrophils < 80, blood procalcitonin < 0.39, synovial calprotectin < 52 mg/L, and neutrophil count < 15,000 all have a negative likelihood ratio < 0.1 and can help to rule out the diagnosis of septic arthritis. In order to help rule in the diagnosis of septic arthritis test findings such as synovial lactate/glucose ratio > 5, synovial lactate > 10, synovial glucose < 1.0, calprotectin > 150 mg/L, synovial leukocyte esterase ++ or +++ with – glucose on dipstick, percent neutrophils > 95, > 50,000 leukocytes, or > 50,000 neutrophils, all in the absence of crystals have likelihood ratios > 10.

## Data Availability

Data is available in tables and figures within the manuscript.
